# *Gynura segetum* induces hepatic sinusoidal obstruction syndrome in mice by impairing autophagy

**DOI:** 10.1590/ACB361104

**Published:** 2022-02-18

**Authors:** Hui Zhang, Shu Jia, Lianyu Jin, Jianzuo Yao MB, Zhihong Shen, Jingyi Wu, Xiaokun Yao, Danwei Chen, Congcong Zhang, Shufang Yu, Ningwei Zhu, Lexiao Jin, Xiaomin Yao

**Affiliations:** 1 Master. Department of Pharmacy - The Second Affiliated Hospital and Yuying Children’s - Hospital of Wenzhou Medical University - Wenzhou Medical University - Wenzhou, China.; 2 PhD. Faculty of Pharmacy - Zhejiang Pharmaceutical College - Ningbo, China.; 3 Bachelor. Infectious Diseases - Ningbo Yinzhou No.2 Hospital - Ningbo, China.; 4 Bachelor. School of Medicine - Ningbo University, China.

**Keywords:** Hepatic Veno-Occlusive Disease, Pyrrolizidine Alkaloids, Liver, Autophagy, Mice

## Abstract

**Purpose:**

To investigate the underlying mechanism of hepatic sinusoidal obstruction syndrome (HSOS) induced by *Gynura segetum* by measuring autophagy in mouse models.

**Methods:**

The model group was administered *G. segetum* (30 g/kg/d) by gavage, while the normal control group was administered an equal volume of saline daily for five weeks. Serum alanine aminotransferase (ALT), aspartate aminotransferase (AST), hepatic histopathological examinations, and Masson staining were performed to evaluate liver injury. Liver intercellular adhesion molecule-1 (ICAM-1) and P-selectin were evaluated by immunohistochemistry. Hepatocellular apoptosis was assessed using the terminal deoxynucleotidyl transferase dUTP nick-end labeling (TUNEL) assay. Protein expression levels of autophagy markers were measured using Western blot analysis.

**Results:**

*Gynura segetum* was found to significantly induce liver injury compared with control mice, as evidenced by the increase of serum transaminases, a decrease in triglyceride levels, and histopathological changes in mice. *Gynura segetum* remarkably induced hepatocellular apoptosis and upregulated the expressions of ICAM-1 and P-selectin and also downregulated the protein expression levels of LC3, Atg12 and cytoplasmic polyadenylation element binding protein.

**Conclusions:**

Our results suggested that *G. segetum* induced liver injury with HSOS, and it was partly due to its ability to impair the autophagy pathway.

## Introduction

Hepatic sinusoidal obstruction syndrome (HSOS), also named hepatic veno-occlusive disease (HVOD), is defined as a non-thrombotic obstruction of the sinusoids without thrombosis or other underlying disorders of the hepatic veins^1^. HSOS can be induced by the consumption of herbal medicines containing pyrrolizidine alkaloids (*Gynura segetum*), hematopoietic cell transplantation, and intense chemotherapy or radiation^2^^-^ 4. The mortality rate of HSOS induced by pyrrolizidine alkaloids is 6-30%, due to the severity of the disease and the absence of effective therapies^5^.

*Gynura segetum*, tusanqi or jusanqi, is a kind of traditional Chinese medicine and it is widely used for pain relief, improving blood circulation, and as an anti-inflammatory agent. However, it contains pyrrolizidine alkaloids that can induce HSOS. Pyrrolizidine alkaloids are mainly present in the roots of plants^6^. Previous studies have demonstrated that pyrrolizidine alkaloids are metabolized by hepatic cytochrome P450 (CYP2B and CYP3A) into dihexyl phthalate esters after being absorbed in the gut^7^. Additionally, small hepatic vessels, particularly the sinusoidal endothelium, have been shown to be damaged by the dihexyl phthalate adducts. Damaged sinusoids contribute partially or completely to the occlusion of small hepatic veins, which eventually leads to HSOS. However, the molecular biological mechanism of HSOS induced by *G. segetum* is still not clear with only a few published reports to date^8^^-^ 10.

Recent studies have indicated that autophagy may disease-dependently participate in the pathogenesis of liver diseases, such as steatosis, liver hepatitis, liver ischemia and reperfusion injury, fibrosis, cirrhosis, and hepatocellular carcinoma^11^^,^ 12. Autophagy is the process through which parts of the cell are degraded in the lysosome. Autophagy includes microautophagy, macroautophagy, and chaperone-mediated autophagy (CMA). Macroautophagy is a stepwise vacuole biogenesis process. The ubiquitin conjugation enzyme 1 (E1) activates ATG12 through a thioester bonding with ATG12. ATG12 then activates the LC3 family of proteins, which are then subsequently cleaved by cysteine protease ATG4 to generate LC3-I. LC3-I then becomes covalently to form the lipidated form of LC3-II via an enzymatic process^13^.

The purposes of the present study were to investigate the underlying mechanisms of *G. segetum* induced HSOS in mice and to identify whether impairment of autophagy contributes to injury.

## Methods

The experiment protocol was approved by Experimental Animal Ethics Committee of Zhejiang Pharmaceutical College.

Male ICR mice weighing 22-24 g were purchased from the Zhejiang Academy of Medical Sciences. This study was performed in compliance with the Animal Research: Reporting of In Vivo Experiments (ARRIVE) guidelines. The experiments were carried out in the experimental center of Faculty of Pharmacy, Zhejiang Pharmaceutical College.

### Reagents

*Gynura segetum* roots were purchased from Anhui Bozhou Pharmaceutical Co. (China) and they were placed in water and soaked for 2 hours, and then boiled for 1.5 hour. The solution was then filtered (filtrate A). Additional water was added to the root and decocted for 1.5 hour using the already mentioned method, and the solution was filtered (filtrate B). Filtrate A and B were then combined, heated, concentrated to 500 mL and stored in the 4°C until required.

Aspartate aminotransferase (AST), alanine aminotransferase (ALT), triglyceride (TG), and cholesterol (CHO) assay kits were obtained from Ningbo Meikang Biological Co. (China). TdT-mediated dUTP nick end labeling (TUNEL) was obtained from Wuhan Boster Biological Engineering Co. (China). Intercellular adhesion molecule-1 (ICAM-1) and P-selectin antibody were purchased from Wuhan Boster Biological Technology (China). BCA protein assay kit was purchased from TianGen Biotech Co. (China). Radioimmunoprecipitation (RIPA) buffer (P0013) was obtained from Beyotime Institute of Biotechnology (China). LC3, Agt12, glyceraldehyde 3-phosphate dehydrogenase (GAPDH), and cytoplasmic polyadenylation element binding protein (CPEB4) antibodies are products of ProteinTech Group, Inc. (United States) and Abcam plc.322 of Cambridge Science Park (United Kingdom). Other chemicals were purchased from the local market.

### Treatments

Animals were randomly divided into two groups with ten mice in each group. For the *G. segetum*-intoxicated group, mice were intragastrically administered 30 g/kg/d concentrated decoction of *G. segetum* every day for five weeks, while mice in the control group were administered an equal volume of vehicle as control. Mice were observed daily and monitored for appetite, hair loss, ascites, activity, and body weight.

At five weeks after *G. segetum* administration, blood samples (collected via the eyeball) and liver tissues were harvested from all animals after 12 h of fasting for subsequent analysis. Liver tissue was rapidly dissected, and two pieces of tissues from the same lobe from each animal were fixed for histopathological analysis. The rest of the liver tissues were snap-frozen in liquid nitrogen for biochemical assays and protein isolation.

### Serum biochemistry

Blood samples at five weeks after *G. segetum* administration were centrifuged to obtain serum and used to measure ALT, AST, TG, and CHO levels. Analytes were measured using the biochemical analyzer (PUZS-300, Beijing Prolong New Technology Co., China) and commercial assay kits, according to the manufacturer’s protocol. The results were calculated based on a standard curve.

### Histopathology analysis

Liver tissues from all mice at five weeks after *G. segetum* administration were fixed with 10% neutral formalin solution and then sectioned. After alcohol gradient dehydration, paraffin embedding, serial sectioning (thickness about 5 μm), hematoxylin and eosin (HE) staining, the sections were visualized for pathological changes under an optical microscope.

Evaluations were performed using a modified scoring system based on DeLeve *et al*.^14^, which included six parameters: sinusoidal hemorrhage, subendothelial hemorrhage of central venules, coagulative necrosis of hepatocytes, endothelial damage of the central venules, subendothelial fibrosis of the central venules, and sinusoidal fibrosis. Hepatic fibrosis was assessed using Masson’s trichrome staining. The slides were photographed using ×100 objective lens to assess histopathological changes. Masson’s trichrome staining was quantified using the Image J software.

### TUNEL assay

Liver tissue sections at five weeks after *G. segetum* administration were examined using *a situ* cell apoptosis detection kit. Briefly, sections were treated with proteinase K for 15 min, rinsed 3×2 min with 0.01 mol/L tris-buffered saline (TBS), and incubated for 2 h at 37°C with TdT and DIG-d-UTP labeling buffer in a humidified chamber. The sections were rinsed for 2 min with 0.01 mol/L TBS buffer and then incubated in blocking buffer for 30 min at room temperature. The sections were then incubated in biotinylated anti-digoxin antibodies (1:100 dilution) for 40 min at 37°C, rinsed for 2 min, three times with 0.01 mol/L TBS buffer. The sections were then stained with streptavidin-fluorescein (FITC) and rinsed five times for 5 min with 0.01 mol/L TBS buffer. Finally, the sections were mounted in antifade solution and analyzed using a confocal laser scanning microscopy. Foci (green) were quantified using the Image J software.

### Immunohistochemistry assay

Formalin-fixed liver samples from all mice were embedded in paraffin and then sliced into 5-μm thick sections. Paraffin sections of liver tissues were analyzed using the indirect immunoperoxidase technique. Briefly, the sections were treated with 0.3% H_2_O_2_ in methanol for 10 min to block endogenous peroxide activity and then incubated with a 300-fold dilution of rabbit anti-mouse ICAM-1 and P-selectin overnight at 4°C. The sections were then incubated with biotinylated goat anti-rabbit IgG as the secondary antibody at 37°C for 15 min followed by 3.30-diaminobenzidine staining. The sections were mounted in antifade solution and analyzed using an immunofluorescent microscope. At last, the expressions were quantified using the Image J software.

### Western blot analysis

Liver tissues were washed with phosphate buffered saline (PBS) and lysed on ice for 30 min in radioimmunoprecipitation (RIPA) buffer (10 mmol/L phosphate buffer pH 7.4, 2 mmol/L EDTA, 0.1% sodium dodecyl sulfate, 150 mmol/L NaCl, 1% sodium deoxycholate, 1% Triton X-100) containing 1 mmol/L sodium orthovanadate and protease inhibitors. After centrifugation at 13,000 rpm for 15 min at 4°C, the supernatant was transferred to a new tube, and protein concentration was determined using the BCA assay.

Western blotting was performed using 40 μg protein lysate. Specific primary antibodies for LC3, Atg12, CPEB4, and secondary antibodies including horseradish peroxidase-conjugated anti-rabbit and anti-goat IgG antibody were used to detect the expression levels of autophagy-related markers. GAPDH was used as an internal loading control. Immunoreactive bands were visualized using the ECL Western Blot Detection System (LAS 400 Mini, General Electric, United States).

### Statistical analysis

All results expressed as mean ± standard deviation (SD) were analyzed by one-way analysis of variance (ANOVA) with the Statistical Package for the Social Sciences (SPSS) 21.0 software. The differences between means were analyzed by Student-Newman-Keuls (SNK) test for multiple comparisons. P value less than 0.05 was considered statistically significant.

## Results

### Gynura segetum induced changes in clinical indices

**Figure 1:**
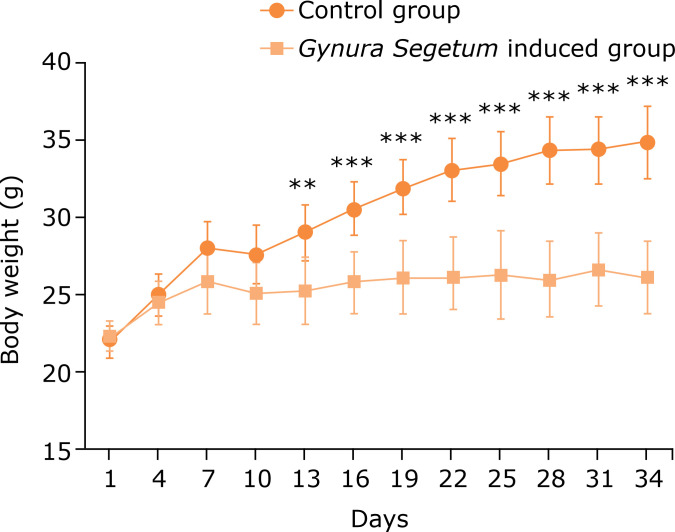
*Gynura segetum* induced the changes of the weight gain in mice (n = 10). Mice were observed daily and monitored for body weight. Compared with the control group, the weight gain of *G. segetum* induced group was slightly smaller.

At five weeks after *G. segetum* administration, the body weight of mice in the *G. segetum* treated group was increased significantly. The abdominal cavity was enlarged, and translucent ascites were observed after laparotomy. Compared to the control group, the weight gain in mice in the *G. segetum* treated group was slightly lower (Fig. 1).

**P < 0.01; ***P < 0.001 *vs*. control group.

### Increase of serum biochemical indices after Gynura segetum administration

**Figure 2:**
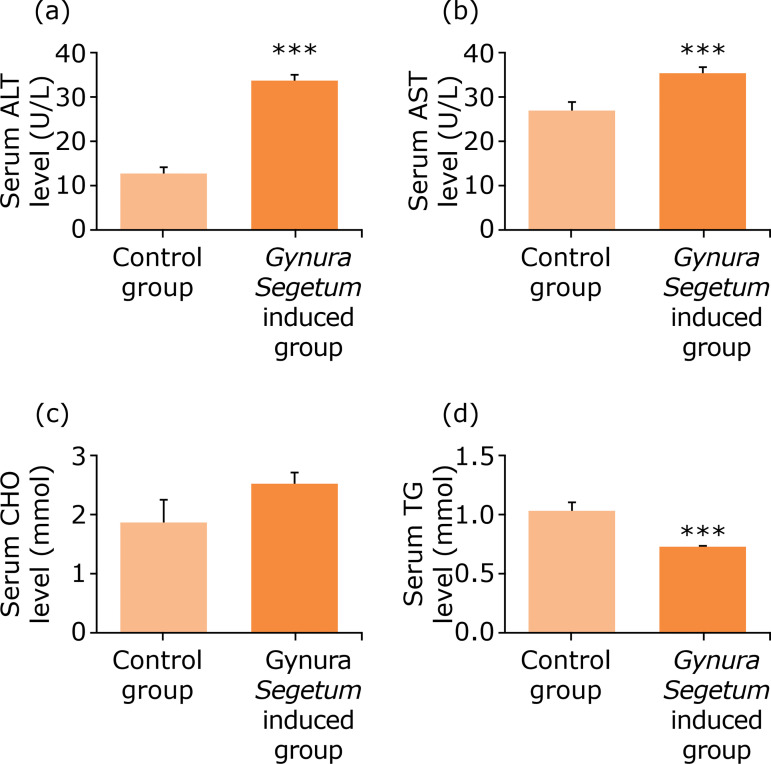
*Gynura segetum* induced the increase of serum biochemical indices in mice (n = 10). Blood samples were obtained at five weeks after *G. segetum* administration: **(a)** serum ALT; **(b)** serum AST; **(c)** serum CHO; **(d)** serum TG.

ALT: alanine aminotransferase; AST: aspartate aminotransferase; CHO: cholesterol; TG: triglyceride; *** P < 0.001 *vs*. control group.

As shown in Fig. 2, compared to the control group, serum ALT and AST levels in the *G. segetum* treated group were significantly increased and 2.5 times and 1.3 time higher compared to the control group, respectively. In addition, the serum TG levels in the *G. segetum* treated group was notably reduced by 37%, while serum CHO levels remained unchanged compared to the control group.

### Gynura segetum induced the changes in histopathology

**Figure 3:**
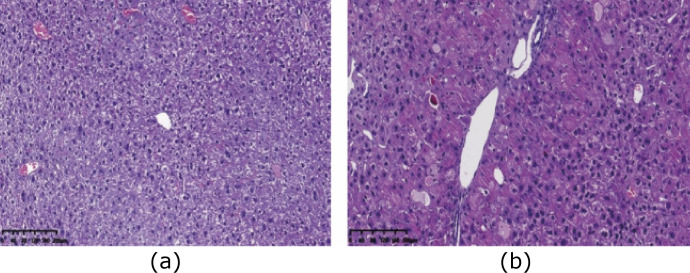
*Gynura segetum* induced the changes of histopathology with hematoxylin and eosin staining in mice (n = 10). Liver specimens were collected at five weeks after *G. segetum* administration, and liver sections were stained with hematoxylin-eosin staining. **(a)** control group; **(b)** *G. segetum*-induced group. Original magnification, ×100.

**Figure 4:**
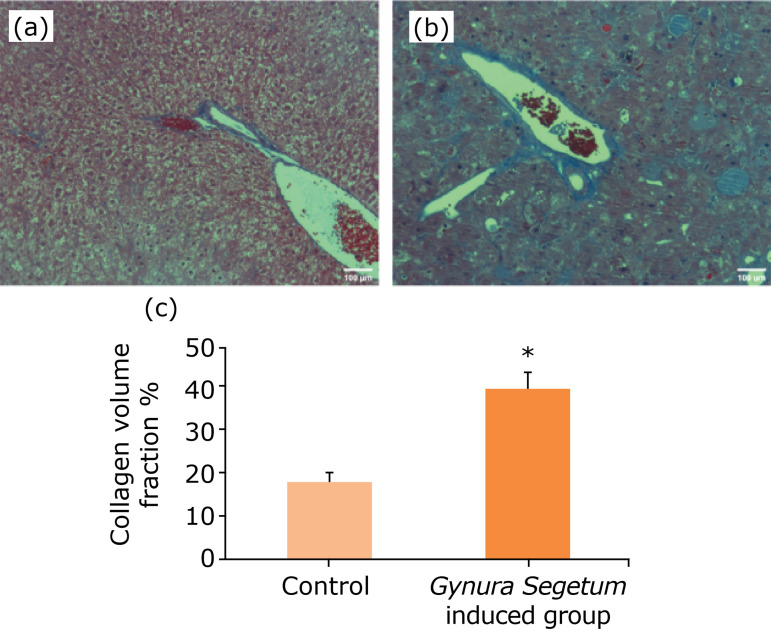
*Gynura segetum* induced the changes of histopathology with Masson staining in mice (n = 10). Liver specimens were collected at five weeks after *G. segetum* administration, and liver sections were stained with Masson staining. Collagen fibers are blue, muscle fibers are red. **(a)** Control group; **(b)** *G. segetum*-induced group. Original magnification ×100.

As shown in Figs. 3 and 4, compared to mice in the control group, liver pathological changes as observed by HE and Masson staining showed liver lobule structure destruction, some hepatocyte nucleus pyknosis, inflammatory cell accumulation in the portal area, fibrosis in some portal areas, fatty degeneration of hepatocytes, severe sinusoidal congestion, and narrowing of hepatic sinusoidal space. In addition, the proliferation of hepatic sinusoidal endothelial and Kupffer cells was observed in mice in the *G. segetum* treated group. The pathological evaluation is shown in Table 1.

**Table 1:** Hepatic sinusoidal obstruction syndrome evaluation for hematoxylin and eosin staining in mice.

**Parameters**	**Control group**	***Gynura segetum-*induced group**
Endothelial injury in the central vein	-	+++
Sinusoidal hemorrhage	-	+++
Coagulative necrosis of hepatocytes	-	+++
Manifold fibrosis	-	+++
Radial arrangement of liver lobules	Normal	Structure disappeared

Gynura segetum *induced hepatocellular apoptosis*

**Figure 5:**
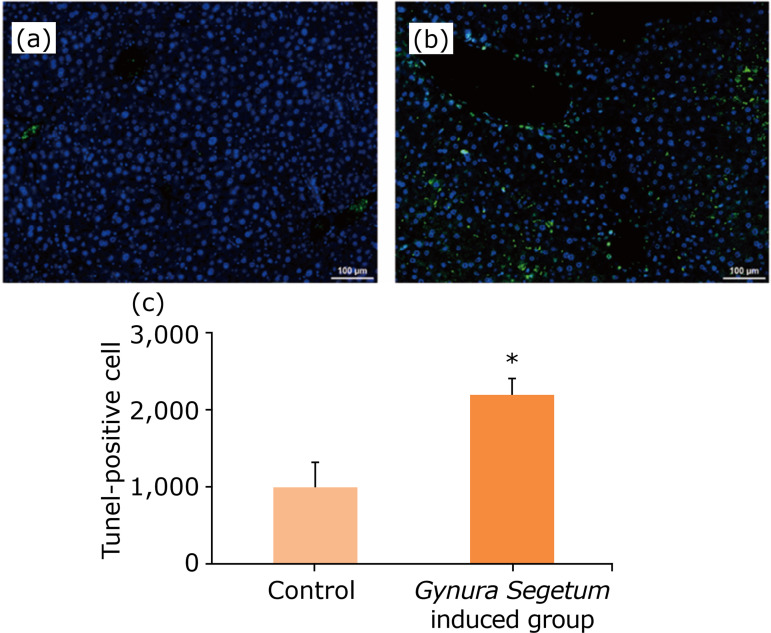
*Gynura segetum* induced hepatocellular apoptosis in mice (TUNEL assay) (n=10). Liver specimens were collected at five weeks after *G. segetum* administration. Foci (green) were quantified by an Image J software. **(a)** control; **(b)** *G. segetum*-induced group. Original magnification ×100.

TUNEL assays were used to assess apoptosis in hepatic tissues. A larger percentage of apoptotic cells was observed in mice administered *G. segetum* compared to control mice at five weeks after *G. segetum* administration (Fig. 5).

*P<0.05 *vs*. control group.

### Increased expression of inflammatory factors after Gynura segetum administration

**Figure 6:**
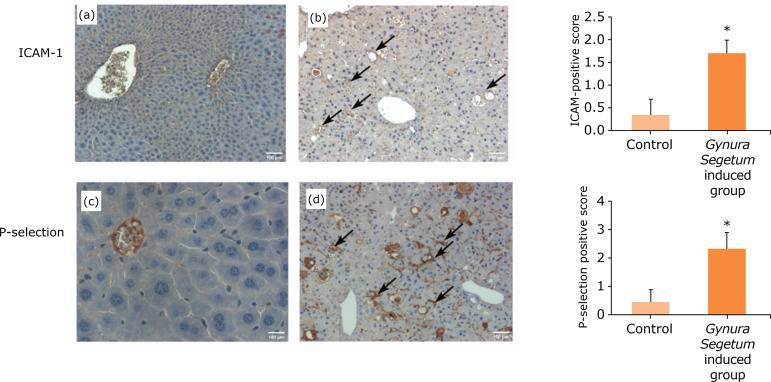
*Gynura segetum* induced the increased expressions of inflammatory factors in mice (n = 10). Liver specimens were collected at five weeks after *G. segetum* administration and detected by immunohistochemistry. The arrow points to high expression. **(a and c)** Control; **(b and d)** *G. segetum*-induced group. Original magnification ×100.

Figure 6 shows the results of immunohistochemical staining for ICAM-1 and P-selectin, which are predominantly expressed in the vascular endothelium. At five weeks after *G. segetum* administration, high expression levels of ICAM-1 and P-selectin in endothelial cells were observed in *G. segetum* treated mice.

ICAM-1: intercellular adhesion molecule-1.

### Gynura segetum downregulated hepatic autophagy-related protein expression levels

**Figure 7:**
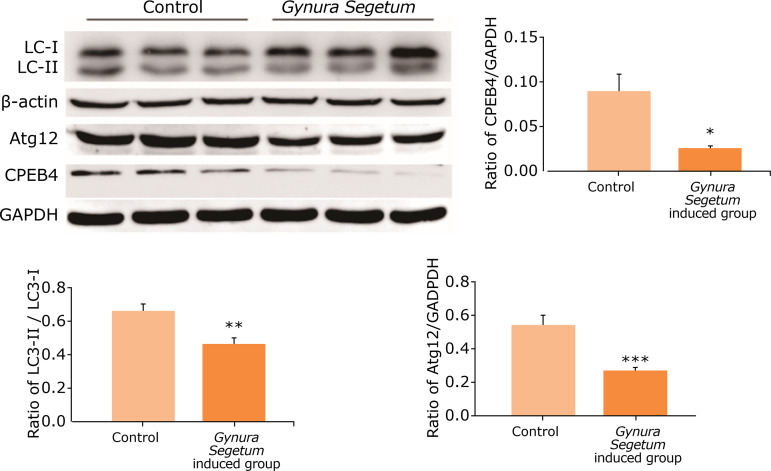
*Gynura segetum* induced hepatic autophagy-related protein expressions in mice (n = 10). Liver tissues were collected at five weeks after *Gynura segetum* administration, and protein expressions (LC3, Atg12 and CPEB4) were analyzed by Western blot.

To investigate the changes of autophagy-related protein expression levels after *G. segetum* administration, several key markers of autophagy–LC3, CPEB4, and Atg12–were measured. As shown in Fig. 7, the protein expression levels of LC3 were markedly downregulated, while Atg12 protein levels were significantly downregulated compared to control mice. Furthermore, CPEB4 levels were remarkably downregulated at five weeks after *G. segetum* administration.

## Discussion

HSOS can be induced by pyrrolizidine alkaloids-containing herbal formulations such as *G. segetum*. The results of our study demonstrated that *G. segetum* administration for five weeks induced HSOS in mice, as evidenced by the increased levels of serum transaminase, the decreased levels of TG, liver pathological changes characterized by inflammatory cell accumulation in the portal area, fibrosis in some portal areas, fatty degeneration of hepatocytes, severe sinusoidal congestion, and the narrowing of hepatic sinusoidal spaces. Additionally, increased proliferation of hepatic sinusoidal endothelial and Kupffer cells was observed.

Two previous studies demonstrated that 18 out of 81 patients (22%) developed HSOS, and the development of HSOS was associated with increased levels of C-reactive protein, indicating that inflammatory response may be associated with HSOS development^15^^,^ 16. In the present study, pathological examination of liver tissues at five weeks after *G. segetum* administration in mice demonstrated a large number of inflammatory cell accumulation in the portal area. Inflammatory responses may be associated with *G. segetum*-induced HSOS. Upregulation of P-selectin and ICAM-1 during experimental murine listeriosis could play an important role in the recruitment of leukocytes, especially to the liver, lymphoid organs, and central nervous system^17^^,^ 18. The role of leukocyte adhesion molecules, such as ICAM-1 and P-selectin, in various organ injuries has been evaluated. The expression levels of ICAM-1 and P-selectin in sinusoidal endothelial cells in the present study were measured using immunohistochemical staining. Our results showed that the expression levels of ICAM-1 and P-selectin in endothelial cells were significantly increased, indicating that HSOS induced by *G. segetum* was related to the increased expression of endothelial inflammatory factors.

In the development and pathogenesis of many liver diseases, apoptosis has been demonstrated to play a key role. Previous *in vitro* and *in vivo* studies have demonstrated that pyrrolizidine alkaloids could lead to hepatotoxicity via apoptosis^19^^,^ 20. Pyrrolizidine alkaloids induce apoptosis by caspase-3 activation, mitochondrial release of cytochrome C, and nuclear fragmentation^21^. Apoptosis was measured using TUNEL assays. In the present study, TUNEL assays demonstrated that apoptosis increased during HSOS induced after *G. segetum* administration.

Autophagy is a catabolic process by which eukaryotic cells eliminate cytosolic materials through vacuole-mediated sequestration and subsequent delivery to lysosomes for degradation. This maintains cellular homeostasis and the integrity of organelles^22^^-^ 24. Autophagy plays a critical role in the regulation of liver physiology and the balance of liver metabolism^25^. The liver is rich in lysosomes and has high levels of metabolic-stress-induced autophagy, which is regulated by levels of hormones and amino acids^26^. Previous studies have shown that liver autophagy contributes to basic hepatic function, such as glycogenolysis, gluconeogenesis, and β-oxidation. This is through selective turnover of specific cargos controlled by a series of transcription factors^27^^,^ 28. To determine whether autophagy is involved in *G. segetum*-induced HSOS, we measured key indicators of autophagy, *i.e.*, LC3 and Atg12 in the present study. Our result demonstrated that *G. segetum* administration induced the downregulation of autophagy markers, LC3, and Atg12. This suggested that autophagy was involved in *G. segetum*-induced HSOS and may contribute to liver injury.

CPEB4 is a member of the cytoplasmic polyadenylation element-binding (CPEB) family of ribonucleic acid (RNA)-binding proteins and part of the autophagy pathway. It regulates mRNA localization and translation by recognizing a cis-acting element, named cytoplasmic polyadenylation element (CPE), which is present in the 3’UTR of target mRNAs^29^^-^ 31. The autophagy promoter Atg12 gene contains a CPE sequence^32^. To evaluate whether CPEB4 is involved in *G. segetum*-induced HSOS, CPEB4 levels were measured using Western blotting. Our results showed that *G. segetum* administration induced the downregulation of CPEB4 protein expression and was consistent with the expression levels of autophagy marker proteins. This suggested that CPEB4 participates in the regulation of HSOS induced by *G. segetum*.

## Conclusion

*Gynura segetum* could induce HSOS in mice and was closely related to the decline of autophagy and the downregulation of CPEB4. Our study provides valuable experimental data for the pathogenesis of HSOS induced by *G. segetum*.

## References

[1] Kan X, Ye J, Rong X, Lu Z, Li X, Wang Y, Yang L, Xu K, Song Y, Hou X. (2016). Diagnostic performance of Contrast-enhanced CT in pyrrolizidine alkaloids-induced hepatic sinusoidal obstructive syndrome. Sci Rep.

[2] Zhang L, Li Q, Makamure J, Zhao D, Liu Z, Zheng C, Liang B. (2021). Transjugular intrahepatic portosystemic shunt for hepatic sinusoidal obstruction syndrome associated with consumption of Gynura segetum. BMC Gastroenterol.

[3] von Salmuth, van der, Bekkers I, van Runnard, Spaanderman MA, Peeters LL, Koek GH. (2020). The role of hepatic sinusoidal obstruction in the pathogenesis of the hepatic involvement in HELLP syndrome: Exploring the literature. Pregnancy Hypertens.

[4] Yang XQ, Ye J, Li X, Li Q, Song YH. (2019). Pyrrolizidine alkaloids-induced hepatic sinusoidal obstruction syndrome: pathogenesis, clinical manifestations, diagnosis, treatment, and outcomes. World J Gastroenterol.

[5] Wang JY, Gao H. (2014). Tusanqi and hepatic sinusoidal obstruction syndrome. J Dig Dis.

[6] Cheng D, Kirk H, Mulder PP, Vrieling K, Klinkhamer PG. (2011). Pyrrolizidine alkaloid variation in shoots and roots of segregating hybrids between Jacobaea vulgaris and Jacobaea aquatica. New Phytol.

[7] Ma J, Xia Q, Fu PP, Lin G (2018). Pyrrole-protein adducts - A biomarker of pyrrolizidine alkaloid-induced hepatotoxicity. J Food Drug Anal.

[8] Yang L, Huo JR, Zhu HY, Chen Z, Wang XY. (2019). The effect of Salvia miltiorrhiza in a mouse model of hepatic sinusoidal obstruction syndrome induced by Gynura segetum. Rev Esp Enferm Dig.

[9] Qiu S, Zhang H, Fei Q, Zhu F, Wang J, Jia X, Chen B. (2018). Urine and plasma metabolomics study on potential hepatoxic biomarkers identification in rats induced by Gynura segetum. J Ethnopharmacol.

[10] Zhang F, Zhou Y, Yang X, Xiong AZ, Wang ZT, Yang L. (2019). Gynura Rhizoma containing pyrrolizidine alkaloids induces the hepatic sinusoidal obstruction syndrome in mice via upregulating fibrosis-related factors. Acta Pharmacol Sin.

[11] Ke PY. (2019). Diverse functions of autophagy in liver physiology and liver diseases. Int J Mol Sci.

[12] Nakazato PCG, Victorino JP, Fina CF, Mendes KDS, Gomes MCJ, Evora PRB, D’Albuquerque LAC, Castro-E-Silva O (2018). Liver ischemia and reperfusion injury. Pathophysiology and new horizons in preconditioning and therapy. Acta Cir Bras.

[13] Mizushima N (2018). A brief history of autophagy from cell biology to physiology and disease. Nat Cell Biol.

[14] DeLeve LD, McCuskey RS, Wang X, Hu L, McCuskey MK, Epstein RB, Kanel GC (1999). Characterization of a reproducible rat model of hepatic veno-occlusive disease. Hepatology.

[15] Jordan K, Pontoppidan P, Uhlving HH, Kielsen K, Burrin DG, Weischendorff S, Christensen IJ, Jørgensen MH, Heilmann C, Sengeløv H, Müller K (2017). Gastrointestinal toxicity, systemic inflammation, and liver biochemistry in allogeneic hematopoietic stem cell transplantation. . Biol Blood Marrow Transplant.

[16] Huang Z, Chen M, Wei M, Lu B, Wu X, Wang Z, Ji L (2019). Liver inflammatory injury initiated by DAMPs-TLR4-MyD88/TRIF-NFkappaB signaling pathway is involved in monocrotaline-induced HSOS. Toxicol Sci.

[17] Ham YM, Song HS, Kwon JE, Jeon H, Baek HJ, Kim CW, Yoon WJ, Choung ES, Kang SC (2019). Effects of fermented Sorghum bicolor L. Moench extract on inflammation and thickness in a vascular cell and atherosclerotic mice model. J Nat Med.

[18] Edwards EE, Thomas SN (2017). P-Selectin and ICAM-1 synergy in mediating THP-1 monocyte adhesion in hemodynamic flow is length dependent. Integr Biol (Camb).

[19] Wang W, Yang X, Chen Y, Ye X, Jiang K, Xiong A, Yang L, Wang Z. (2020). Seneciphylline, a main pyrrolizidine alkaloid in Gynura japonica, induces hepatotoxicity in mice and primary hepatocytes via activating mitochondria-mediated apoptosis. J Appl Toxicol.

[20] Waizenegger J, Braeuning A, Templin M, Lampen A, Hessel-Pras S. (2018). Structure-dependent induction of apoptosis by hepatotoxic pyrrolizidine alkaloids in the human hepatoma cell line HepaRG: Single versus repeated exposure. Food Chem Toxicol.

[21] Ji L, Chen Y, Liu T, Wang Z. (2008). Involvement of Bcl-xL degradation and mitochondrial-mediated apoptotic pathway in pyrrolizidine alkaloids-induced apoptosis in hepatocytes. Toxicol Appl Pharmacol.

[22] Ueno T, Komatsu M. (2017). Autophagy in the liver: functions in health and disease. Nat Rev Gastroenterol Hepatol.

[23] Ke PY (2019). Diverse functions of autophagy in liver physiology and liver diseases. Int J Mol Sci.

[24] Li X, He S, Ma B. (2020). Autophagy and autophagy-related proteins in cancer. Mol Cancer.

[25] Madrigal-Matute J, Cuervo AM (2016). Regulation of liver metabolism by autophagy. Gastroenterology.

[26] Chi HC, Tsai CY, Tsai MM, Yeh CT, Lin KH (2019). Molecular functions and clinical impact of thyroid hormone-triggered autophagy in liver-related diseases. J Biomed Sci.

[27] Allaire M, Rautou PE, Codogno P, Lotersztajn S. (2019). Autophagy in liver diseases: Time for translation?. J Hepatol.

[28] Panzitt K, Fickert P, Wagner M (2021). Regulation of autophagy by bile acids and in cholestasis - CholestoPHAGY or CholeSTOPagy. Biochim Biophys Acta Mol Basis Dis.

[29] Pascual R, Segura-Morales C, Omerzu M, Bellora N, Belloc E, Castellazzi CL, Reina O, Eyras E, Maurice MM, Millanes-Romero A, Mendez R. (2020). mRNA spindle localization and mitotic translational regulation by CPEB1 and CPEB4. RNA.

[30] Tseng CS, Chao HW, Huang HS, Huang YS. (2017). Olfactory-experience- and developmental-stage-dependent control of CPEB4 regulates c-Fos mRNA translation for granule cell survival. Cell Rep.

[31] Arasaki Y, Li M, Akiya T, Nozawa I, Ezura Y, Hayata T. (2020). The RNA-binding protein Cpeb4 is a novel positive regulator of osteoclast differentiation. Biochem Biophys Res Commun.

[32] Rojas-Ríos P, Chartier A, Pierson S, Séverac D, Dantec C, Busseau I, Simonelig M. (2015). Translational control of autophagy by orb in the Drosophila Germline. Dev Cell.

